# Genome-Wide Association Study of Genetic Variants in LPS-Stimulated IL-6, IL-8, IL-10, IL-1ra and TNF-α Cytokine Response in a Danish Cohort

**DOI:** 10.1371/journal.pone.0066262

**Published:** 2013-06-18

**Authors:** Margit Hørup Larsen, Anders Albrechtsen, Lise Wegner Thørner, Thomas Werge, Thomas Hansen, Ulrik Gether, Eva Haastrup, Henrik Ullum

**Affiliations:** 1 Department of Clinical Immunology, Copenhagen University Hospital, Copenhagen, Denmark; 2 Centre for Bioinformatics, University of Copenhagen, Copenhagen, Denmark; 3 Institute of Biological Psychiatry, Mental Health Centre Sct. Hans, Copenhagen University Hospital, Roskilde, Denmark; 4 Molecular Neuropharmacology and Genetics Laboratory, University of Copenhagen, Copenhagen, Denmark; INSERM, France

## Abstract

**Background:**

Cytokine response plays a vital role in various human lipopolysaccharide (LPS) infectious and inflammatory diseases. This study aimed to find genetic variants that might affect the levels of LPS-induced interleukin (IL)-6, IL-8, IL-10, IL-1ra and tumor necrosis factor (TNF)-α cytokine production.

**Methods:**

We performed an initial genome-wide association study using Affymetrix Human Mapping 500 K GeneChip® to screen 130 healthy individuals of Danish descent. The levels of IL-6, IL-8, IL-10, IL-1ra and TNF-α in 24-hour LPS-stimulated whole blood samples were compared within different genotypes. The 152 most significant SNPs were replicated using Illumina Golden Gate® GeneChip in an independent cohort of 186 Danish individuals. Next, 9 of the most statistical significant SNPs were replicated using PCR-based genotyping in an independent cohort of 400 Danish individuals. All results were analyzed in a combined study among the 716 Danish individuals.

**Results:**

Only one marker of the 500 K Gene Chip in the discovery study showed a significant association with LPS-induced IL-1ra cytokine levels after Bonferroni correction (*P*<10^−7^). However, this SNP was not associated with the IL-1ra cytokine levels in the replication dataset. No SNPs reached genome-wide significance for the five cytokine levels in the combined analysis of all three stages.

**Conclusions:**

The associations between the genetic variants and the LPS-induced IL-6, IL-8, IL-10, IL-1ra and TNF-α cytokine levels were not significant in the meta-analysis. This present study does not support a strong genetic effect of LPS-stimulated cytokine production; however, the potential for type II errors should be considered.

## Introduction

Lipopolysaccharide (LPS) is an amphiphilic molecule present at high concentrations on the outer membrane of Gram-negative bacteria. LPS is of crucial importance, as it protects cell membranes from chemical attack and helps stabilizing the overall membrane structure of the Gram-negative bacteria [1]. In mammals, LPS is an endotoxin which stimulates and induces a response from the mammalian innate immune system. The mechanism by which LPS is brought to the surface of responsive immune cells and initiates inflammatory responses begins with binding to lipid binding proteins (LBP) in human serum. After binding to LBP, LPS will be transferred to CD14 which is expressed as a membrane-bound glycoprotein on the cell surface of monocytes, macrophages and neutrophiles. The binding of LPS to CD14 leads to the recruitment of the toll-like receptor 4 (TLR4) molecule which is able to mediate signals which activate a broad range of genes, including those for IL-1ra (anti-inflammatory), IL-6 (pro-inflammatory), IL-8 (pro-inflammatory) and IL-10 (predominantly anti-inflammatory) and TNF-α (pro-inflammatory); all are important mediators in the regulation of the immune response and inflammatory process [2].

Association studies between IL-1ra, IL-6, IL-8, IL-10 and TNF-α levels and polymorphisms in the genes encoding these cytokines have been reported with inconsistent results [3]. However, it has been suggested that the production and levels of cytokines may be influenced by genes outside their primary coding loci [4] for example by regulatory single-nucleotide polymorphisms (rSNP) [5]. A few genome-wide association studies (GWAS) considering association of heritable components in the variation of cytokine production have been reported [3], [4], [5]. We conducted a GWAS of the following five cytokines; IL-1ra, IL-6, IL-8, IL-10 and TNF-α, in population-based LPS-stimulated blood samples of healthy Danish individuals.

## Materials and Methods

### Samples and Study Design

**Figure 1:**
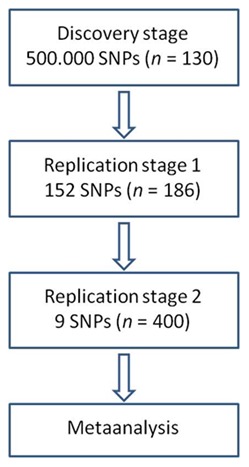
Study design illustrating the genome-wide association (GWA) study approach.

The study design is summarized in figure 1.

Individuals included in this study were healthy Danish blood donors (382 women and 334 men) from Copenhagen University Hospital, Rigshospitalet, Denmark. Blood samples were obtained by using the remaining blood from the side portion collected at blood donation. Data collected from each participant included sex and a blood sample. The study was approved by the Central Medical Scientific Ethics Committee of Denmark (KF 01–024/01 and KF 11303898). Oral and written informed consent was obtained from the first included participants (*n* = 130, discovery phase). The remaining samples included in the study (replication phase 1 and 2) were all spare blood handled anonymously and could thus be used without collection of informed consent form according to Danish regulations (act no 593 of 14 June 2011).

### LPS-stimulation and Cytokine Assessment

All blood samples were diluted 1∶4 with RPMI, and then 2 ml of each sample was stimulated with 20 µl of LPS (100 µg/ml) (Sigma-Aldrich, Copenhagen, Denmark). Afterwards, the samples were incubated at 37°C with 5% CO_2_ for 24 hours, followed by centrifugation at 2500 crp for 10 min. The supernatants were harvested by gently pipetting and subsequently frozen and stored at -80°C.Concentrations of the cytokines IL-1ra, IL-6, IL-8, IL-10 and TNF-α were quantified by a Luminex 100 platform (Luminex Corporation, Austin, TX) using R&D Human Fluorokine® MAP assays (R&D Systems®, Cary, NC). Analyses were performed according to the manufacturer’s suggested protocol.

### Genotyping

We performed a genome-wide association study in 130 Danish individuals using the Affymetrix Human Mapping 500 K GeneChip® to search for genetic variants that influence LPS-stimulated cytokine production (discovery stage). Next, 152 candidate SNPs from the 500 k GeneChip analysis were investigated further in 186 Danish individuals using the Illumina Golden Gate® GeneChip (first replication stage). Results from the first two stages were analyzed in a combined study, from which 9 identified SNPs with top-ranked *P*-values were investigated in 400 Danish individuals (second replication stage) using the Kbiosciences PCR genotyping system (KASPar) as an in-house service at KBiosciences. Finally, we analyzed the same data for all three stages in a meta-analysis to identify associations between gene variants and IL-6, IL-8, IL-10, IL-1ra and TNF-α cytokine response related to LPS stimulation. As the genotype and phenotype data for the current study will not be publicly available, we kindly request readers to contact corresponding author for any further information on the dataset.

### Quality Control and SNPs Chosen for Replication

Inbreeding coefficients were estimated on the X chromosome in order to determine gender. All individuals inferred gender matched the reported gender. The inbreeding coefficients were also estimated based on the autosome using a method of moment estimator. Extreme values of this coefficient are either a sign of inbreeding or genotype errors. All individuals had an inbreeding coefficient close to 0 (-0.06 to 0.06). Signs of batch effects or population structure were evaluated using both multi dimensional scaling based on the identical state matrix which showed no signs of clustering. Pairwise relatedness was estimated between all subjects and close relationships (1 cousins or closer) were removed from the analysis. Genotyping errors were determined using 6 duplicated samples which showed a discordance rate of 0.3% using the BRLMM (Bayesian Robust Linear Model with Mahalanobis Distance Classifier) call confidence score above 0.5. In the discovery stage only SNPs with a *P*-value equal to or less than 0.0001 to the LPS trait were selected for further analysis. Then SNPs with a minor allele frequency (MAF) of less than 0.05 and SNPs where Hardy-Weinberg equilibrium were rejected with a *P*-value less than 0.001 were removed. Afterwards, the SNPs from the discovery stage were trimmed so that no pair of SNPs (using 10 SNP window) were in high linkage disequilibrium (R^2^>0.8) where the less associated SNPs were removed.

### Study Design and Statistical Analysis

Cytokine levels were evaluated between men and women by analysis of covariance (ANCOVA).

Normality of the IL-6, IL-8, IL-10, IL-1ra and TNF-α quantitative phenotypes were assessed using descriptive statistics and distribution plots (histograms and q-q plots). None of the cytokines were normally distributed; therefore, values were natural log-transformed prior to genome-wide analysis. The analysis was done in PLINK statistical software by using linear regression models under an additive genetic model with adjustment for sex. The 152 top-scoring SNPs from the initial discovery cohort were genotyped further in 186 healthy individuals (replication stage 1) followed by a combined analysis. Then, 9 of the 152 SNPs which reached the most significant values in a combined analysis of discovery phase and replication stage 1 were genotyped in 400 individuals (replication stage 2). Afterwards, results from SNPs typed in all of the three stages were combined in a meta-analysis, from which estimated heterogeneity variance and forest plots from each stage and the combined analysis were generated. In order to compensate for the number of tests performed, the obtained *P*-values needed to pass the Bonferroni adjustment and permutation testing. Bonferroni adjustment was simply done by dividing 0.05 by 5·10^5^ tests giving a *P*-value of 1×10^−7^. Adjustment for multiple testing was also performed using a permutation procedure. This was done by permuting the trait labels randomly between samples. The highest test statistic across the whole genome was saved in each iteration in order to obtain an empirical null distribution that corrects for the multiple testing. Power calculation was performed using Bioconductor’s GeneticsDesign showing that minor allele frequencies of 50%, 20% and 10% have 80% power to detect a difference between groups of 0.45 × SD, 0.87 × SD and 0.99 × SD respectively.

## Results

Cytokine levels of all the study subjects after 24 hour of LPS stimulation are presented in table 1. There were no differences in IL-6, IL-8, IL-10 and TNF-α cytokine levels between men and women; however, women had significantly higher IL-1ra levels than men (*P* = 0.002), therefore sex is included in the general linear model.

**Table 1:** The untransformed cytokine levels (pg/ml) measured in supernatants after 24 hours LPS-stimulation from the healthy individuals included in this genome-wide association study.

Cytokine	Mean (pg/ml) ± SD			*P*-value
	Overall (*n* = 716)	Men (*n* = 334)	Women (*n* = 382)	
**IL-1ra**	35870±11472	34422±11149	37135±11616	0.002
**IL-6**	16398±5778	16697±5709	16138±5833	0.20
**IL-8**	11382±6255	11761±5904	11052±6537	0.13
**IL-10**	374±302	367±249	382±343	0.51
**TNF-α**	3021±1742	3141±1663	2916±1804	0.08

*P*-values show significance of mean concentration difference between men and women by two-sample T-test of means.

### GWA Analysis (Discovery Stage)

**Figure 2:**
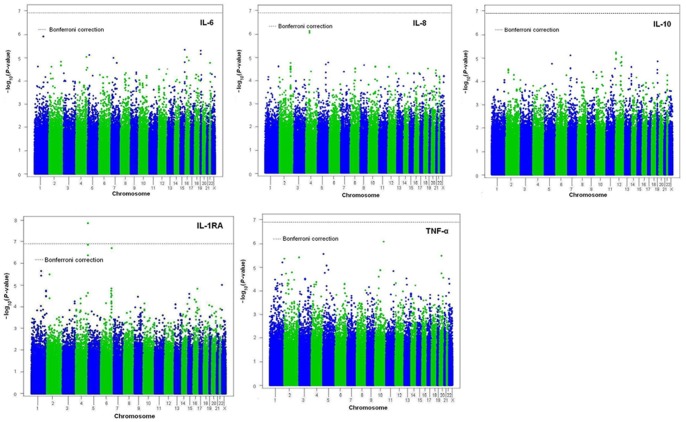
Manhattan plots showing *P*-values of association of each of the SNPs in the analysis with LPS stimulated plasma cytokine levels. SNPs are plotted on the X-axis to their position on each chromosome against cytokine levels on the Y-axis (shown as –log_10_ *P*-value).

Figure 2 shows Manhattan plots of the *P*-values of the Affymetrix Human Mapping 500 K GeneChip in the discovery phase; results are presented for the sex-combined (*n* = 130) analysis. One of these SNPs (rs12512853, *P* = 1.3×10^−8^) on chromosome 4 was associated with the produced IL-1ra levels with a genome-wide significant *P*-value (*P*<1×10^−7^) after adjustment for sex. This significance is maintained through a Bonferroni correction, figure 2 (IL-1ra). No SNP reached genome-wide significance for the other four cytokine production levels. Yet, significance was not evident after the rs12512853 IL-1ra SNP was corrected for multiple testing using the permutation procedure (*P* = 1.6×10^−6^). We selected the top 152 most associated LPS-stimulated SNPs for further analysis and confirmation in a new sample, replication stage 1 (*n = *186). The top 152 most associated LPS-stimulated SNPs selected for the first replication stage are presented in Table S1.

### Replication Analysis and Combined Analysis (Replication Stage 1 and 2)

**Figure 3:**
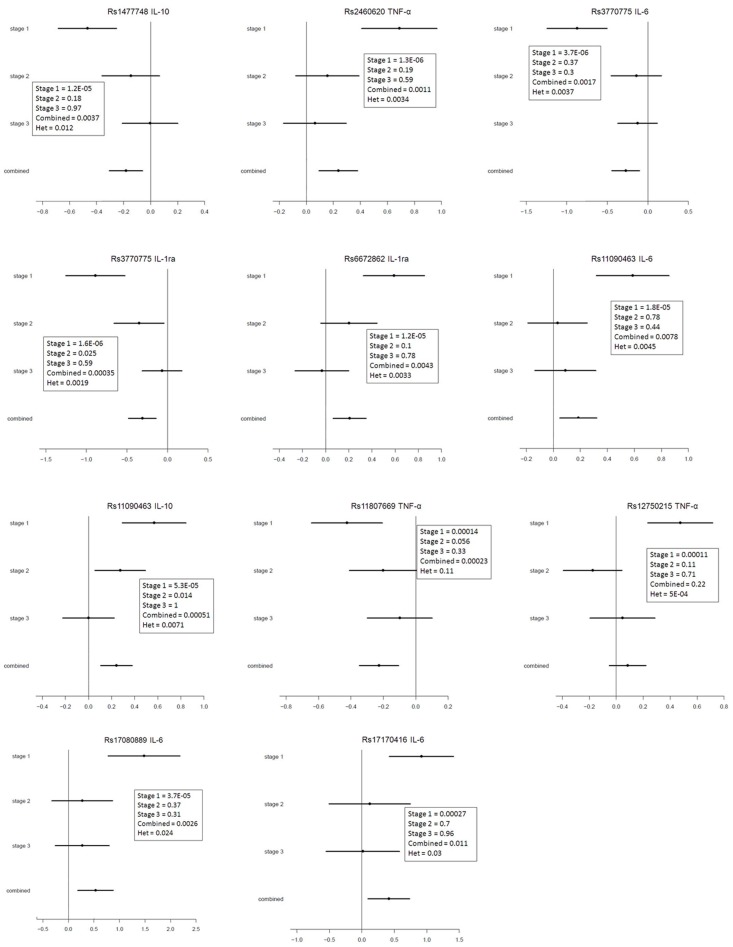
Forest plots of meta-analysis of each selected SNP on the cytokine production. The x-axis represents the estimated effect sizes in mean differences. The horizontal lines show the stage-specific estimated effect sizes and the corresponding 95% confidence intervals of the natural logarithmic mean differences. The solid vertical line at 0 means no difference in effect size.

None of the 152 best associated SNPs from the discovery stage were statistically significant after adjustment for multiple testing in replication stage 1. We chose the top 9 SNPs from the first replication stage to be further analyzed in a second replication stage. Association analysis of the 9 most interesting SNPs, solely based on the *P*-values, was done for the three individual stages and for the combined stages; the results are shown in table 2 and figure 3. The forest plots in figure 3 allow a visual assessment of the amount of variation between the results of the different stages, which shows that results in the current study are heterogeneous. The results from the meta-analysis show that after being corrected for multiple testing none of the SNPs associated with cytokine levels after 24 hour of LPS treatment.

**Table 2:** Top 9 SNPs showing the highest evidence of association with IL-6, IL-8, IL-10, IL-1ra and TNF-α LPS stimulated plasma levels among healthy individuals with the most associated *P*-values <5×10^−5^ in the discovery stage or *P*-values <0.05 in the first replication stage (*n* = 716).

Cytokine	SNP	Chr	Position	*P*-value discovery	*P*-value replication 1	*P*-value replication 2	*P*-value combined	Het
**IL-10**	rs1477748	12	75738812	**2.1·10^−5^**	0.18	0.97	0.0037	0.012
**TNF-α**	rs2460620	15	44085750	**1.3·10^−6^**	0.19	0.59	0.0011	0.0034
**IL-6**	rs3770775	2	37033711	**3.7·10^−6^**	0.37	0.3	0.0017	0.0037
**IL-1ra**	rs3770775	2	37033711	**1.6·10^−6^**	**0.025**	0.59	3.5·10^−4^	0.0019
**IL-1ra**	rs6672862	1	236850434	**1.2·10^−5^**	0.1	0.78	0.0043	0.0033
**IL-6**	rs11090463	22	25707569	**1.8·10^−5^**	0.78	0.44	0.0078	0.0045
**IL-10**	rs11090463	22	25707569	**5.3·10^−5^**	**0.014**	1.0	5.1·10^−4^	0.0071
**TNF-α**	rs11807669	1	94842702	**1.4·10^−4^**	**0.056**	0.33	2.3·10^−4^	0.11
**TNF-α**	rs12750215	1	87927757	**1.1·10^−4^**	0.11	0.71	0.22	5·10^−4^
**IL-6**	rs17080889	13	19837136	**3.7·10^−5^**	0.37	0.31	0.0026	0.024
**IL-6**	rs17170416	7	33781320	**2.7·10^−4^**	0.7	0.96	0.011	0.03

Chromosomal positions are from NCBI build 36 (hg 18).

The results from the discovery stage could not be replicated in any of the further analysis done in the replication stages and meta-analysis. No association between genetic variants and LPS-stimulated IL-6, IL-8, IL-10 and TNF-α cytokine levels was seen in healthy Danish blood donors.

## Discussion

In general, when performing GWAS with quantitative measurements, the potential for type I errors can be high. However, Bonferroni is a very conservative correction and the association of a given genetic variant on cytokine level might be too small to reach genome wide significance when comparing 500,000 tests. As for any empirical research, the statistical power for GWAS is of critical importance. As an example, we had 80% power to detect an effect size of 0.87×SD between groups with a minor allele frequency of 20%. According to our sample size, minor or modest effects of genetic variants on the produced cytokine levels might be missed in the current study.In the discovery stage we found significant SNPs of whom one SNP also passed the Bonferroni correction. This SNP did not reach significant level after permutation-based correction for multiple testing; this might be because the phenotype is not completely normally distributed and the statistics might be inflated giving artificially low *P*-values. This distorts the type I error for the asymptotic test and makes the permutation procedure less powerful. However, none of these SNPs could be replicated in the further stages. From the meta-analysis illustrated in the forest plot we see some wide confidence intervals for all of the studies. This indicates that we have a low power in the three stages. As the mean values are logarithmic, some differences in effect sizes can be missed. An effect difference of 0.2 on the x-axis on the forest plot (figure 3) is actually a 1.2 fold difference (е^0.2^ = 1.2) in mean cytokine level. Therefore some of the SNPs which show the same direction of effect on mean values, even though not significant, is relevant to be further investigated in a new set-up.

Among healthy individuals the cytokine production is known to be influenced by factors such as age, BMI, and ethnicity. Our study population only included Caucasian individuals; however, we did not obtain information about their parental and grandparental ancestry. Furthermore, we did not adjust for age in the analysis. These factors might have an impact on the results obtained.

However, the mentioned factors are just speculative in this current study and should be further investigated in determining the possible effect on the cytokine production.

## Supporting Information

Table S1 Top 152 SNPs showing the highest evidence of association with IL-6, IL-8, IL-10, IL-1RA and TNF-α LPS stimulated plasma levels from the discovery phase among Danish individuals (*n* = 130). Chromosomal positions are from NCBI build 36 (hg 18).
(DOCX)
