# Supplementary material for: Genome-Wide Association Study of Genetic Variants in LPS-Stimulated IL-6, IL-8, IL-10, IL-1ra and TNF-α Cytokine Response in a Danish Cohort
Source: PLoS One. 2013 Jun 18;8(6):e66262. doi: 10.1371/journal.pone.0066262 (PMC3688878; doi:10.1371/journal.pone.0066262)
Supplement: Table S1 — Top 152 SNPs showing the highest evidence of association with IL-6, IL-8, IL-10, IL-1RA and TNF-α LPS stimulated plasma levels from the discovery phase among Danish individuals (n = 130). Chromosomal positions are from NCBI build 36 (hg 18). (DOCX) [file pone.0066262.s001.docx]

**Supplementary table 1.**

| **SNP** | **Chr** | **Position** | **Cytokine** | **MAF** | **HWE** | **Alleles** | ***P*-value** |
| --- | --- | --- | --- | --- | --- | --- | --- |
| **rs12512853** | 4 | 187990630 | IL-1ra | 0.48 | 0.40 | A/G | 1.30E-08 |
| **rs17081491** | 6 | 149130006 | IL-1ra | 0.31 | 0.31 | C/T | 5.70E-08 |
| **rs13105379** | 4 | 187991865 | IL-1ra | 0.40 | 0.30 | C/T | 1.60E-07 |
| **rs7663862** | 4 | 55454959 | IL-8 | 0.06 | 1.00 | A/G | 7.80E-07 |
| **rs2460620** | 15 | 44085750 | TNF-α | 0.23 | 1.00 | C/T | 1.30E-06 |
| **rs3770775** | 2 | 37033711 | IL-1ra | 0.11 | 1.00 | G/T | 2.80E-06 |
| **rs1330372** | 1 | 168293867 | IL-1ra | 0.33 | 0.11 | C/T | 2.90E-06 |
| **rs6136819** | 20 | 19631718 | TNF-α | 0.48 | 1.00 | A/G | 3.20E-06 |
| **rs6471859** | 8 | 61340216 | IL-6 | 0.44 | 1.00 | C/G | 3.60E-06 |
| **rs11683693** | 2 | 241155342 | TNF-α | 0.14 | 0.77 | A/C | 3.60E-06 |
| **rs12911109** | 15 | 92208225 | IL-6 | 0.15 | 0.16 | C/T | 5.00E-06 |
| **rs682765** | 19 | 15562078 | IL-6 | 0.18 | 0.34 | C/T | 5.90E-06 |
| **rs7549968** | 1 | 221744288 | TNF-α | 0.08 | 0.61 | C/T | 5.90E-06 |
| **rs17289150** | 12 | 85269795 | IL-10 | 0.11 | 0.71 | A/G | 6.40E-06 |
| **rs3782633** | 12 | 5666214 | IL-10 | 0.44 | 0.56 | A/G | 7.30E-06 |
| **rs3779238** | 7 | 33730314 | IL-10 | 0.12 | 0.17 | C/T | 7.60E-06 |
| **rs2153110** | 6 | 145285032 | IL-1ra | 0.26 | 0.27 | A/G | 7.70E-06 |
| **rs8045506** | 16 | 23659147 | IL-6 | 0.49 | 1.00 | C/T | 8.40E-06 |
| **rs7719524** | 5 | 20895398 | IL-6 | 0.47 | 1.00 | C/T | 9.20E-06 |
| **rs9351508** | 6 | 66272757 | IL-1ra | 0.33 | 0.75 | C/G | 9.50E-06 |
| **rs224956** | 5 | 81739502 | TNF-α | 0.06 | 0.15 | C/T | 1.20E-05 |
| **rs7091374** | 10 | 72409032 | TNF-α | 0.06 | 0.46 | A/G | 1.20E-05 |
| **rs17170416** | 7 | 33781320 | IL-6 | 0.06 | 0.42 | A/G | 1.20E-05 |
| **rs220872** | 11 | 114771575 | TNF-α | 0.45 | 0.39 | A/G | 1.40E-05 |
| **rs3917538** | 7 | 94775829 | IL-6 | 0.23 | 0.56 | A/G | 1.40E-05 |
| **rs731945** | 19 | 18428039 | IL-10 | 0.36 | 0.76 | C/T | 1.40E-05 |
| **rs2168608** | 16 | 69240195 | IL-6 | 0.50 | 0.48 | C/T | 1.50E-05 |
| **rs17006964** | 2 | 119298975 | TNF-α | 0.12 | 0.01 | C/T | 1.50E-05 |
| **rs1477748** | 12 | 75738812 | IL-10 | 0.41 | 0.07 | A/G | 1.60E-05 |
| **rs16840018** | 2 | 204257085 | IL-6 | 0.48 | 1.00 | A/G | 1.70E-05 |
| **rs2377524** | 9 | 77962279 | IL-8 | 0.35 | 0.34 | A/T | 1.70E-05 |
| **rs7565347** | 2 | 20385435 | TNF-α | 0.05 | 1.00 | C/T | 1.70E-05 |
| **rs10957702** | 8 | 75306307 | IL-6 | 0.36 | 0.36 | C/T | 1.70E-05 |
| **rs880890** | 2 | 187892745 | IL-8 | 0.33 | 0.75 | C/T | 1.80E-05 |
| **rs17731499** | 5 | 167697178 | IL-8 | 0.16 | 0.12 | G/T | 1.80E-05 |
| **rs3759880** | 15 | 42835943 | IL-1ra | 0.11 | 0.46 | A/G | 1.80E-05 |
| **rs11090463** | 22 | 25707569 | IL-6 | 0.24 | 0.84 | C/T | 1.80E-05 |
| **rs10517763** | 4 | 163483208 | IL-1ra | 0.34 | 0.42 | A/G | 1.90E-05 |
| **rs4846249** | 1 | 216194417 | TNF-α | 0.06 | 0.46 | A/G | 2.00E-05 |
| **rs2717512** | 2 | 36922824 | IL-10 | 0.48 | 1.00 | C/T | 2.10E-05 |
| **rs30530** | 5 | 132136240 | IL-8 | 0.15 | 0.38 | C/G | 2.10E-05 |
| **rs4862742** | 4 | 188054297 | IL-1ra | 0.49 | 0.57 | C/T | 2.10E-05 |
| **rs17080889** | 13 | 19837136 | IL-6 | 0.48 | 1.00 | C/T | 2.10E-05 |
| **rs1434781** | 8 | 69229347 | IL-8 | 0.39 | 0.55 | G/T | 2.10E-05 |
| **rs6988935** | 8 | 75374629 | IL-6 | 0.37 | 0.36 | A/C | 2.10E-05 |
| **rs7995838** | 13 | 112309830 | IL-10 | 0.30 | 0.74 | C/T | 2.20E-05 |
| **rs1903995** | 10 | 53485680 | TNF-α | 0.46 | 0.67 | A/C | 2.30E-05 |
| **rs4738438** | 8 | 75319043 | IL-6 | 0.29 | 0.73 | C/T | 2.40E-05 |
| **rs2049638** | 2 | 34808299 | IL-1ra | 0.30 | 0.87 | C/G | 2.50E-05 |
| **rs12140458** | 1 | 235343646 | IL-8 | 0.42 | 0.47 | G/T | 2.50E-05 |
| **rs964292** | 20 | 52250445 | TNF-α | 0.22 | 0.28 | A/G | 2.60E-05 |
| **rs3770775** | 2 | 37033711 | IL-6 | 0.11 | 1.00 | G/T | 2.60E-05 |
| **rs2801527** | 10 | 90894056 | IL-8 | 0.28 | 0.59 | C/T | 2.70E-05 |
| **rs10841879** | 12 | 8592284 | IL-6 | 0.37 | 1.00 | C/T | 2.70E-05 |
| **rs2724588** | 12 | 11754082 | IL-8 | 0.37 | 0.65 | C/T | 2.70E-05 |
| **rs231821** | 2 | 204381054 | IL-6 | 0.47 | 1.00 | A/G | 2.80E-05 |
| **rs2882203** | 2 | 187990575 | IL-8 | 0.37 | 0.88 | A/T | 2.90E-05 |
| **rs929525** | 18 | 57247745 | IL-8 | 0.28 | 1.00 | A/G | 3.10E-05 |
| **rs6672862** | 1 | 236850434 | IL-1ra | 0.22 | 1.00 | A/G | 3.10E-05 |
| **rs12750215** | 1 | 87927757 | TNF-α | 0.29 | 0.22 | A/G | 3.20E-05 |
| **rs1114337** | 2 | 161624989 | TNF-α | 0.16 | 0.01 | C/T | 3.30E-05 |
| **rs1118699** | 11 | 127296128 | IL-10 | 0.44 | 1.00 | C/G | 3.30E-05 |
| **rs11891079** | 2 | 54219798 | TNF-α | 0.19 | 0.82 | A/G | 3.30E-05 |
| **rs204043** | 16 | 13125675 | IL-1ra | 0.20 | 0.66 | A/G | 3.40E-05 |
| **rs7206252** | 16 | 17691548 | IL-6 | 0.30 | 0.09 | A/T | 3.60E-05 |
| **rs17211953** | 4 | 172863355 | IL-6 | 0.05 | 0.43 | C/G | 3.60E-05 |
| **rs6128940** | 20 | 58898810 | IL-8 | 0.08 | 0.61 | A/G | 3.70E-05 |
| **rs4383087** | 15 | 92254413 | IL-6 | 0.22 | 0.53 | A/T | 4.00E-05 |
| **rs13275576** | 8 | 129358294 | IL-6 | 0.08 | 1.00 | G/T | 4.00E-05 |
| **rs2214873** | 7 | 41785436 | IL-8 | 0.14 | 1.00 | A/T | 4.50E-05 |
| **rs7684846** | 4 | 68049140 | IL-1ra | 0.18 | 0.02 | C/G | 4.70E-05 |
| **rs1490338** | 1 | 216218974 | TNF-α | 0.07 | 0.60 | A/T | 4.70E-05 |
| **rs11068687** | 12 | 116740880 | IL-8 | 0.22 | 0.69 | A/C | 4.70E-05 |
| **rs474030** | 20 | 56202318 | IL-8 | 0.44 | 1.00 | C/T | 4.90E-05 |
| **rs234653** | 1 | 183115396 | TNF-α | 0.45 | 1.00 | C/T | 4.90E-05 |
| **rs17007814** | 3 | 2130262 | IL-6 | 0.21 | 0.67 | A/G | 4.90E-05 |
| **rs10080589** | 6 | 136561217 | TNF-α | 0.49 | 1.00 | A/G | 5.00E-05 |
| **rs8056064** | 16 | 81344554 | IL-8 | 0.14 | 0.03 | A/G | 5.00E-05 |
| **rs898955** | 4 | 38380749 | TNF-α | 0.49 | 1.00 | C/G | 5.10E-05 |
| **rs7220491** | 17 | 2981856 | IL-8 | 0.41 | 0.37 | C/T | 5.10E-05 |
| **rs8177539** | 14 | 76857512 | IL-8 | 0.07 | 0.61 | C/T | 5.10E-05 |
| **rs17148800** | 4 | 71263170 | TNF-α | 0.12 | 0.32 | A/G | 5.10E-05 |
| **rs7917378** | 10 | 103692724 | IL-6 | 0.16 | 0.43 | A/C | 5.10E-05 |
| **rs535914** | 1 | 236829721 | IL-1ra | 0.31 | 1.00 | C/T | 5.20E-05 |
| **rs5753976** | 22 | 31049204 | IL-6 | 0.42 | 0.47 | C/G | 5.40E-05 |
| **rs13427635** | 2 | 21545800 | IL-6 | 0.07 | 0.01 | A/G | 5.40E-05 |
| **rs2099595** | 2 | 128981476 | IL-10 | 0.19 | 0.50 | A/G | 5.50E-05 |
| **rs2065997** | 9 | 102086992 | IL-6 | 0.46 | 0.06 | A/G | 5.50E-05 |
| **rs3783950** | 14 | 80518035 | TNF-α | 0.49 | 0.57 | C/G | 5.60E-05 |
| **rs12468667** | 2 | 40545708 | TNF-α | 0.48 | 1.00 | C/G | 5.70E-05 |
| **rs4723331** | 7 | 33778454 | IL-10 | 0.18 | 0.31 | A/G | 5.70E-05 |
| **rs664617** | 1 | 225364311 | TNF-α | 0.46 | 1.00 | C/T | 5.70E-05 |
| **rs4896767** | 6 | 145352783 | IL-1ra | 0.21 | 0.52 | A/G | 5.70E-05 |
| **rs516594** | 11 | 94260305 | IL-10 | 0.31 | 0.32 | G/T | 5.80E-05 |
| **rs6784029** | 3 | 189462427 | TNF-α | 0.13 | 0.75 | C/T | 5.90E-05 |
| **rs2209158** | 1 | 110596541 | TNF-α | 0.45 | 0.57 | A/C | 6.00E-05 |
| **rs6450226** | 5 | 53946883 | TNF-α | 0.13 | 0.53 | G/T | 6.00E-05 |
| **rs7767039** | 6 | 153160905 | IL-10 | 0.18 | 0.22 | C/T | 6.10E-05 |
| **rs10004442** | 4 | 38383189 | TNF-α | 0.49 | 1.00 | A/G | 6.30E-05 |
| **rs2075416** | 19 | 37173138 | IL-6 | 0.30 | 0.62 | C/T | 6.40E-05 |
| **rs2669139** | 10 | 5834174 | IL-10 | 0.49 | 1.00 | C/T | 6.50E-05 |
| **rs7860550** | 9 | 25685174 | TNF-α | 0.49 | 1.00 | C/T | 6.50E-05 |
| **rs11090463** | 22 | 25707569 | IL-10 | 0.24 | 0.84 | C/T | 6.60E-05 |
| **rs2205505** | 21 | 21907276 | IL-8 | 0.06 | 1.00 | A/C | 6.70E-05 |
| **rs5753394** | 22 | 29653900 | IL-8 | 0.28 | 0.60 | C/G | 6.80E-05 |
| **rs6602745** | 10 | 6576558 | IL-10 | 0.14 | 1.00 | C/T | 6.80E-05 |
| **rs12407003** | 1 | 58712222 | IL-8 | 0.10 | 0.70 | A/G | 6.80E-05 |
| **rs859058** | 1 | 95140237 | TNF-α | 0.39 | 0.76 | C/T | 7.00E-05 |
| **rs9964798** | 18 | 19841320 | IL-6 | 0.27 | 1.00 | C/T | 7.10E-05 |
| **rs1877219** | 3 | 142448273 | TNF-α | 0.23 | 0.43 | A/G | 7.20E-05 |
| **rs12049547** | 1 | 236842238 | IL-1ra | 0.26 | 0.19 | A/C | 7.30E-05 |
| **rs7950942** | 11 | 131939578 | IL-8 | 0.33 | 0.15 | C/T | 7.30E-05 |
| **rs12673613** | 7 | 67072319 | IL-6 | 0.28 | 0.37 | A/G | 7.30E-05 |
| **rs1728397** | 16 | 84965714 | IL-1ra | 0.47 | 1.00 | A/G | 7.40E-05 |
| **rs3917550** | 7 | 94772509 | IL-6 | 0.15 | 0.57 | C/T | 7.40E-05 |
| **rs7756667** | 6 | 133323713 | TNF-α | 0.48 | 1.00 | A/G | 7.50E-05 |
| **rs834238** | 1 | 148633093 | TNF-α | 0.39 | 0.77 | G/T | 1.40E-05 |
| **rs2158951** | 7 | 43733623 | IL-10 | 0.22 | 0.21 | C/T | 7.70E-05 |
| **rs6024378** | 20 | 53769588 | IL-1ra | 0.43 | 1.00 | A/G | 7.70E-05 |
| **rs12359510** | 10 | 9446646 | IL-1ra | 0.29 | 0.30 | C/T | 7.70E-05 |
| **rs2073020** | 6 | 53903236 | IL-6 | 0.22 | 0.42 | A/G | 7.90E-05 |
| **rs9323895** | 14 | 93409883 | IL-10 | 0.10 | 0.70 | A/G | 7.90E-05 |
| **rs9898954** | 17 | 65726365 | IL-8 | 0.47 | 1.00 | A/G | 7.90E-05 |
| **rs35386797** | 2 | 119291520 | TNF-α | 0.12 | 0.17 | C/T | 7.90E-05 |
| **rs13031942** | 2 | 2456650 | IL-8 | 0.19 | 1.00 | C/T | 8.00E-05 |
| **rs17132419** | 11 | 73257737 | IL-10 | 0.45 | 1.00 | A/G | 8.00E-05 |
| **rs4915215** | 1 | 199343835 | IL-6 | 0.41 | 0.56 | C/T | 8.10E-05 |
| **rs803137** | 5 | 132160546 | IL-8 | 0.23 | 0.69 | A/G | 8.10E-05 |
| **rs13073417** | 3 | 3307441 | IL-6 | 0.14 | 1.00 | A/G | 8.20E-05 |
| **rs1277217** | 1 | 109159707 | TNF-α | 0.49 | 1.00 | C/T | 8.40E-05 |
| **rs11807669** | 1 | 94842702 | TNF-α | 0.39 | 0.18 | C/T | 8.50E-05 |
| **rs4689443** | 4 | 6480893 | IL-1ra | 0.21 | 0.08 | C/T | 8.50E-05 |
| **rs9548688** | 13 | 38692731 | IL-8 | 0.31 | 0.07 | A/C | 8.70E-05 |
| **rs2872398** | 13 | 30333076 | IL-1ra | 0.39 | 0.10 | G/T | 8.80E-05 |
| **rs7004004** | 8 | 61347567 | IL-6 | 0.49 | 0.67 | A/T | 8.80E-05 |
| **rs2058469** | 18 | 8143997 | IL-6 | 0.48 | 1.00 | A/G | 8.90E-05 |
| **rs12410954** | 1 | 229310379 | IL-10 | 0.16 | 0.79 | A/C | 8.90E-05 |
| **rs17069905** | 13 | 77935360 | IL-10 | 0.34 | 0.06 | A/G | 9.00E-05 |
| **rs11160417** | 14 | 97372096 | IL-1ra | 0.26 | 0.71 | C/T | 9.00E-05 |
| **rs10935425** | 3 | 142479100 | TNF-α | 0.45 | 0.32 | A/G | 9.10E-05 |
| **rs4241679** | 4 | 67942477 | IL-1ra | 0.34 | 0.21 | A/T | 9.20E-05 |
| **rs9493150** | 6 | 132315684 | IL-1ra | 0.30 | 0.24 | C/G | 9.20E-05 |
| **rs17033172** | 2 | 45269745 | IL-8 | 0.10 | 0.41 | C/T | 9.20E-05 |
| **rs17105914** | 10 | 87619194 | IL-8 | 0.42 | 0.61 | G/T | 9.20E-05 |
| **rs4607937** | 1 | 56042031 | IL-8 | 0.20 | 0.50 | C/T | 9.30E-05 |
| **rs2267355** | 22 | 35347836 | IL-8 | 0.07 | 0.25 | A/G | 9.30E-05 |
| **rs7008686** | 8 | 28154639 | IL-1ra | 0.43 | 0.39 | C/T | 9.30E-05 |
| **rs9898649** | 17 | 78530594 | IL-8 | 0.11 | 0.72 | C/T | 9.30E-05 |
| **rs11688605** | 2 | 28982922 | IL-6 | 0.22 | 1.00 | C/T | 9.60E-05 |
| **rs1125908** | 12 | 45073338 | IL-1ra | 0.44 | 1.00 | A/G | 9.80E-05 |
| **rs9410375** | 9 | 90634443 | IL-6 | 0.13 | 0.03 | C/T | 9.80E-05 |
| **rs7097308** | 10 | 4313923 | IL-6 | 0.48 | 1.00 | C/T | 9.80E-05 |
| **rs4440084** | 3 | 167315035 | IL-8 | 0.47 | 1.00 | C/G | 9.90E-05 |
| **rs2226690** | 22 | 25119906 | IL-10 | 0.20 | 0.51 | C/T | 1.00E-04 |
